# Medial closing wedge distal femoral varus osteotomy alters the stress distribution pattern of the patellofemoral joint: An evaluation using computed tomography osteoabsorptiometry

**DOI:** 10.1002/jeo2.70689

**Published:** 2026-04-20

**Authors:** Masanari Hamasaki, Eiji Kondo, Koji Iwasaki, Yuki Suzuki, Masatake Matsuoka, Tomohiro Onodera, Daisuke Momma, Masayuki Inoue, Kazunori Yasuda, Tomonori Yagi, Norimasa Iwasaki

**Affiliations:** ^1^ Department of Orthopedic Surgery, Faculty of Medicine and Graduate School of Medicine Hokkaido University Sapporo Japan; ^2^ Centre for Sports Medicine Hokkaido University Hospital Sapporo Japan; ^3^ Department of Functional Reconstruction for the Knee Joint, Faculty of Medicine Hokkaido University Sapporo Japan; ^4^ Department of Orthopaedic Surgery NTT East Japan Sapporo Hospital Sapporo Japan; ^5^ Department of Orthopaedic Surgery Yagi Orthopaedic Hospital Sapporo Japan

**Keywords:** CT osteoabsorptiometry, distal femoral varus osteotomy, knee, osteoarthritis, patellofemoral joint, stress distribution

## Abstract

**Purpose:**

This study aimed to (1) assess changes in subchondral bone density distribution across patellofemoral (PF) joint before and after medial closing wedge‐distal femoral varus osteotomy (MCW‐DFVO) and (2) determine correlation between PF alignment and changes in bone density distribution.

**Methods:**

This retrospective study enroled patients who underwent MCW‐DFVO for symptomatic isolated lateral compartment osteoarthritis (OA), spontaneous osteonecrosis of the knee and lateral femoral condyle cartilage injury with valgus alignment from 2016 to 2022. Clinical and radiological assessments were conducted preoperatively and at the final follow‐up. The final follow‐up referred to the last documented visit at which outcome measures were available. The radiological quadriceps angle (rQ angle) was measured using computed tomography (CT). The distribution of subchondral bone density on trochlear and patella was examined using CT osteoabsorptiometry. The lateral ratio was calculated as the proportion of high‐density areas (HDAs) in the lateral compartments relative to the total HDAs across compartments of trochlea and patella. The paired Student's *t* test and Pearson's correlation analysis were used to test for significance (*p* = 0.05).

**Results:**

Seventeen knees (17 patients; mean age, 48 years) were included. Following MCW‐DFVO, the mean postoperative Lysholm score significantly improved at the final follow‐up (mean, 29.2 months; range, 14–65 months). The mean lateral ratio of the trochlea and patella notably declined from 69% to 50% and 69% to 56%, respectively (*p* = 0.004 and *p* = 0.041). Changes in trochlear lateral ratio were significantly correlated with changes in the hip–knee–ankle angle, mechanical axis, rQ angle and lateral shift ratio (*p* = 0.027, *p* = 0.031, *p* = 0.024 and *p* = 0.008, respectively).

**Conclusion:**

MCW‐DFVO induced a redistribution of HDA from lateral to medial PF articular surface. Moreover, degree of PF alignment correction post‐MCW‐DFVO was linked to shifts in HDA distribution. MCW‐DFVO alters PF joint stress distribution by reducing lateral compartment loading, supporting its use in valgus knees with PF mal‐tracking or overload.

**Level of Evidence:**

Level IV, case series.

## Graphical abstract

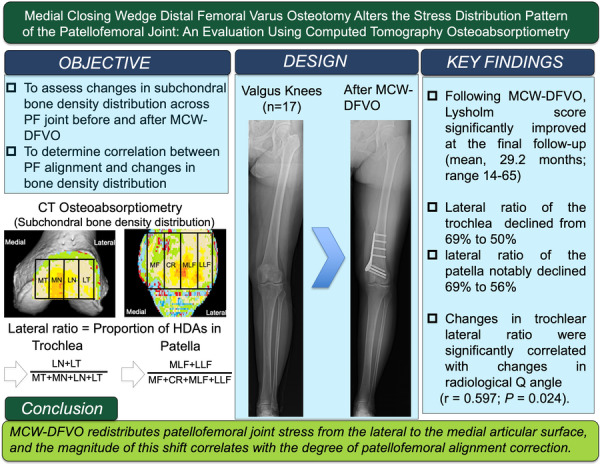

**Abbreviations**
ACI: autologous chondrocyte implantation
BMD: bone mineral density
CD ratio: Caton–Deschamps
CT: computed tomography
DFVO: distal femoral varus osteotomy
FT: femorotibial
HDAs: high‐density areas
HKA angle: hip–knee–ankle angle
HU: Hounsfield units
ICCs: intraclass correlation coefficients
JOA: Japanese Orthopaedic Association
KL: Kellgren–Lawrence
lateral ratio: proportion of HDA in the lateral compartment relative to the total HDA
LLF: lateral portion of the lateral facet
LN: the lateral notch
LSR: lateral shift ratio
LT: lateral trochlea
MA: mechanical axis
MCW‐DFVO: medial closing wedge
MF: medial facet
mLDFA: mechanical lateral distal femoral angle
MLF: medial portion of the lateral facet
MN: medial notch
MPFL: medial patellofemoral ligament
MT: medial trochlea
OA: osteoarthritis
PF: patellofemoral
rQ‐angle: radiological quadriceps angle
SONK: spontaneous osteonecrosis of the knee
TT‐TG distance: tibial tuberosity–trochlear groove
VMO: vastus medialis oblique
%HDA: percentage of HDA

## INTRODUCTION

Femorotibial (FT) osteoarthritis (OA) with valgus knee deformity is associated with an increased incidence of patellofemoral (PF) OA [4, 12, 25]. Previous studies reported that medial closing wedge‐distal femoral varus osteotomy (MCW‐DFVO) can be an effective concomitant procedure for young and middle‐aged patients with focal chondral defects and FT OA in lateral compartment and PF OA [1, 31, 32]. Nevertheless, the specific alterations in stress distribution across the PF articular surface following MCW‐DFVO remain unclear, and in vivo studies characterizing PF joint stress distribution are limited.

To evaluate the effect of MCW‐DFVO on the actual loading condition of the PF joint, the distribution of the subchondral bone mineral density (BMD) is thought to reflect the cumulative stress distribution over the joint surface; because of the varying degrees of mineralization over the surface of a joint, a materialized field of stress is observed [27]. Computed tomography (CT)–osteoabsorptiometry enables in vivo assessment of long‐term stress distribution by quantifying subchondral BMD in individual joints [28]. Previous studies have shown that the magnitude of alignment correction after MCW‐DFVO is closely linked to the shifts in high‐density area (HDA) distribution in the FT joint, reflecting changes in stress distribution [11].

This study aimed to (1) assess changes in subchondral bone density distribution across PF joint before and after MCW‐DFVO and (2) determine correlation between PF alignment and changes in bone density distribution. Accordingly, we hypothesized that (1) MCW‐DFVO reduces subchondral bone density on the lateral PF articular surface in valgus knees and (2) changes in bone density distribution correlate with leg and PF alignment.

## METHODS

### Study design

This retrospective study was approved by our institutional review board, and written informed consent was obtained from all patients at the time of their initial treatment and used clinical data retrospectively. This retrospective study enroled consecutive patients who underwent MCW‐DFVO for symptomatic isolated lateral compartment OA, spontaneous osteonecrosis of the knee (SONK) or cartilage injury of the lateral femoral condyle with valgus alignment from June 2016 to May 2022. This period was selected because MCW‐DFVO was consistently performed using the same surgical method and locking plate fixation from 2016 onward. All eligible patients during this period agreed to participate. All procedures were performed by a senior orthopaedic surgeon (E.K.) with over 25 years of experience in knee surgery. Indications for MCW‐DFVO included moderate valgus alignment (hip–knee–ankle [HKA] angle > 5°), no medial FT joint OA (Kellgren–Lawrence [KL] grade [18] ≥ 2) and PF OA of grade > 3. Exclusion criteria encompassed medial knee OA (KL grade ≥ 2) or medial knee pain, a follow‐up period of at least 1 year, loss of knee extension >15°, range of knee motion <130°, cruciate ligament insufficiency or varus/valgus instability <10°, medial meniscal damage or previous surgery on the affected knee. Patients undergoing concurrent surgery (lateral release, medial PF ligament reconstruction, tibial tuberosity realignment) affecting articular cartilage in the PF and FT joints were also excluded. Postoperative CT was performed as part of our institution's standard clinical protocol following MCW‐DFVO to allow precise objective evaluation of osteotomy alignment, hardware placement, PF joint morphology and subchondral BMD. Patients who did not undergo implant removal after MCW‐DFVO were excluded because the locking plate can affect the CT values due to metal artefact, and finite element analysis has demonstrated a stress‐shielding effect of locking plates on adjacent bone [19]. Patients undergoing double‐level osteotomies or concurrent procedures affecting articular cartilage were also excluded. Clinical and radiological assessments were conducted preoperatively and at the final follow‐up. The final follow‐up referred to the last documented visit at which outcome measures were available.

### Preoperative planning

**Figure 1:**
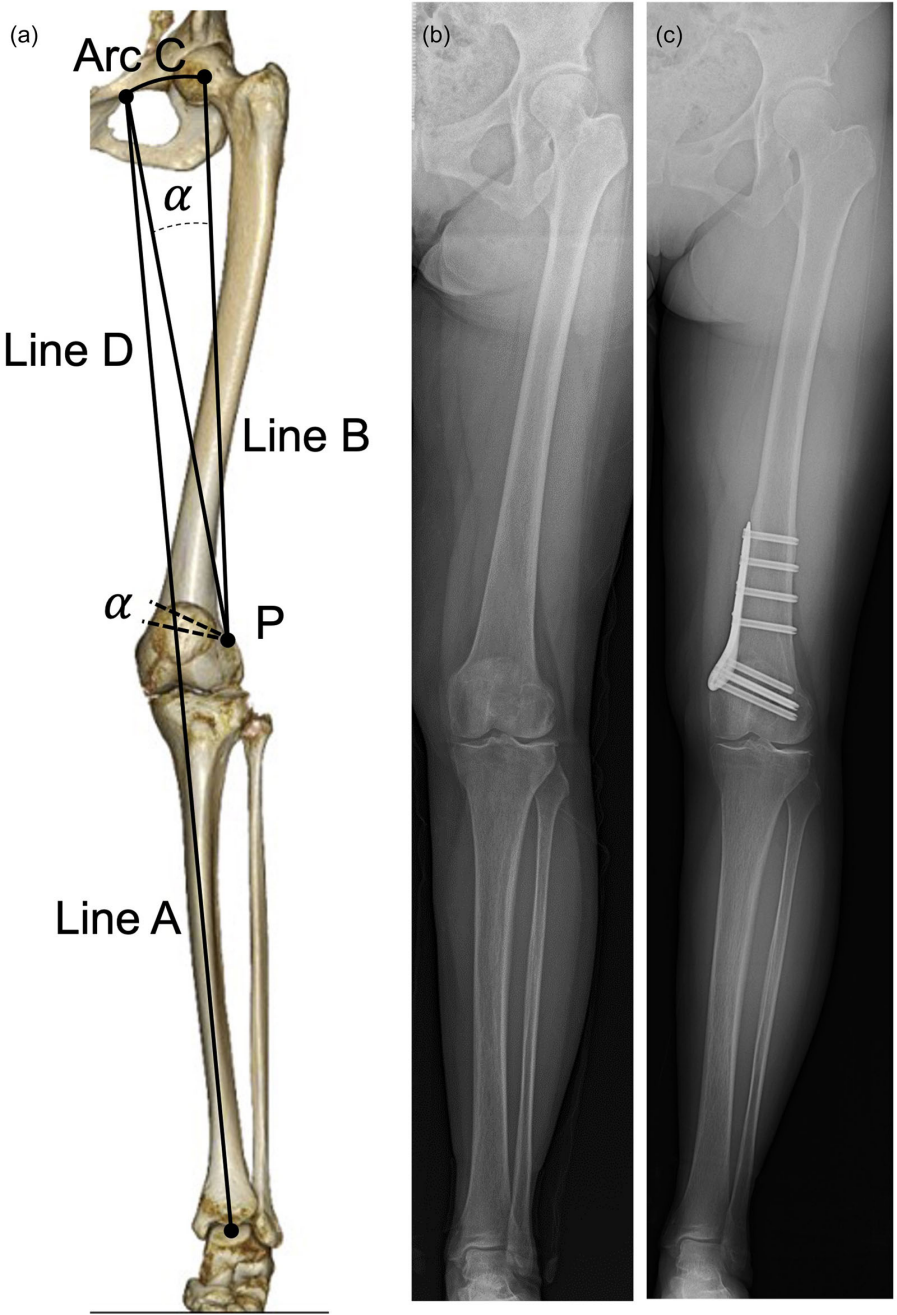
Preoperative planning and pre‐ and postoperative standing (full‐length lower‐limb anteroposterior radiographs for MCW‐DFVO). (a) Preoperative planning. (b) Preoperative standing. (c) Postoperative standing. MCW‐DFVO, medial closing wedge‐distal femoral varus osteotomy.

Preoperative planning was performed using standing full‐length anteroposterior radiographs of the lower limb to determine the correction angle. The Miniaci method [26], as previously reported [15, 29], was applied. Briefly, line A was drawn from the centre of the talar dome through the medial intercondylar eminence of the tibia, corresponding to a mechanical axis (MA) percentage of 42.5% and an HKA angle of 2°, to reduce the risk of medial compartment OA in active patients (Figure 1) [34, 35]. Line B was then drawn from the hinge point (P), located proximal to the upper border of the lateral femoral condyle between the lateral femoral cortex and the contour of the condyle, to the femoral head's centre, with its length recorded. An Arc C, with Radius B, was then drawn from Point P to intersect Line A. Line D was drawn from Point P to the intersection of Lines A and C. The angle between Lines B and D represented the MCW correction angle required to restore lower‐limb alignment. Double‐level osteotomy was considered for valgus knees with a preoperatively anticipated joint line obliquity of >5° [17].

### Surgical procedure of MCW‐DFVO

Each patient underwent diagnostic arthroscopy immediately before MCW‐DFVO. Standard anterolateral and anteromedial parapatellar portals were used to confirm isolated lateral compartment OA, SONK or cartilage injury of the lateral FT joint. The MCW‐DFVO procedure was performed as previously described [15, 29]. Briefly, a 7‐cm antero‐medial longitudinal incision was made on the medial thigh. To protect the neurovascular structures, a radiolucent retractor (Osferionbiomaterials) was positioned between the femur and the detached posterior structures. An ascending biplanar osteotomy was then performed under fluoroscopic guidance. The lateral hinge point was identified at the lateral‐proximal section of the lateral femoral condyle on anterior‐posterior fluoroscopic images [30], and a guidewire was inserted toward this point. Additional guidewires were inserted from a point 4 cm proximal to the medial epicondyle toward the hinge point, with two distal guidewires placed at the predetermined correction angle using a wedge‐cutting guide. The initial ascending cut was made 10 mm below the anterior femoral cortex using a thin oscillating saw and chisel, followed by a transverse cut along the guidewires. After removal of the bone wedge, osteotomy site was carefully closed. Fixation was achieved using an anatomical locking compression plate (TriS medial DFO plate system; Osferionbiomaterials). Four screws were then inserted into the distal femoral condyle, compression was applied across the osteotomy using a compression screw, and four additional screws secured the femoral shaft.

### Postoperative rehabilitation

Postoperative rehabilitation commenced with continuous passive motion exercises 1 week following the operation. Transitioning from partial to full weight‐bearing was permitted between 3 and 6 weeks post‐surgery.

### Clinical and radiological evaluations

#### Clinical evaluation

Patients were assessed preoperatively and at the final follow‐up using the Lysholm knee score [23]. Minimal clinically important difference (MCID) achievement was analysed for the Lysholm score [33, 38]. The MCID thresholds were 10.2 points for the Lysholm score.

#### Radiological evaluation

**Figure 2:**
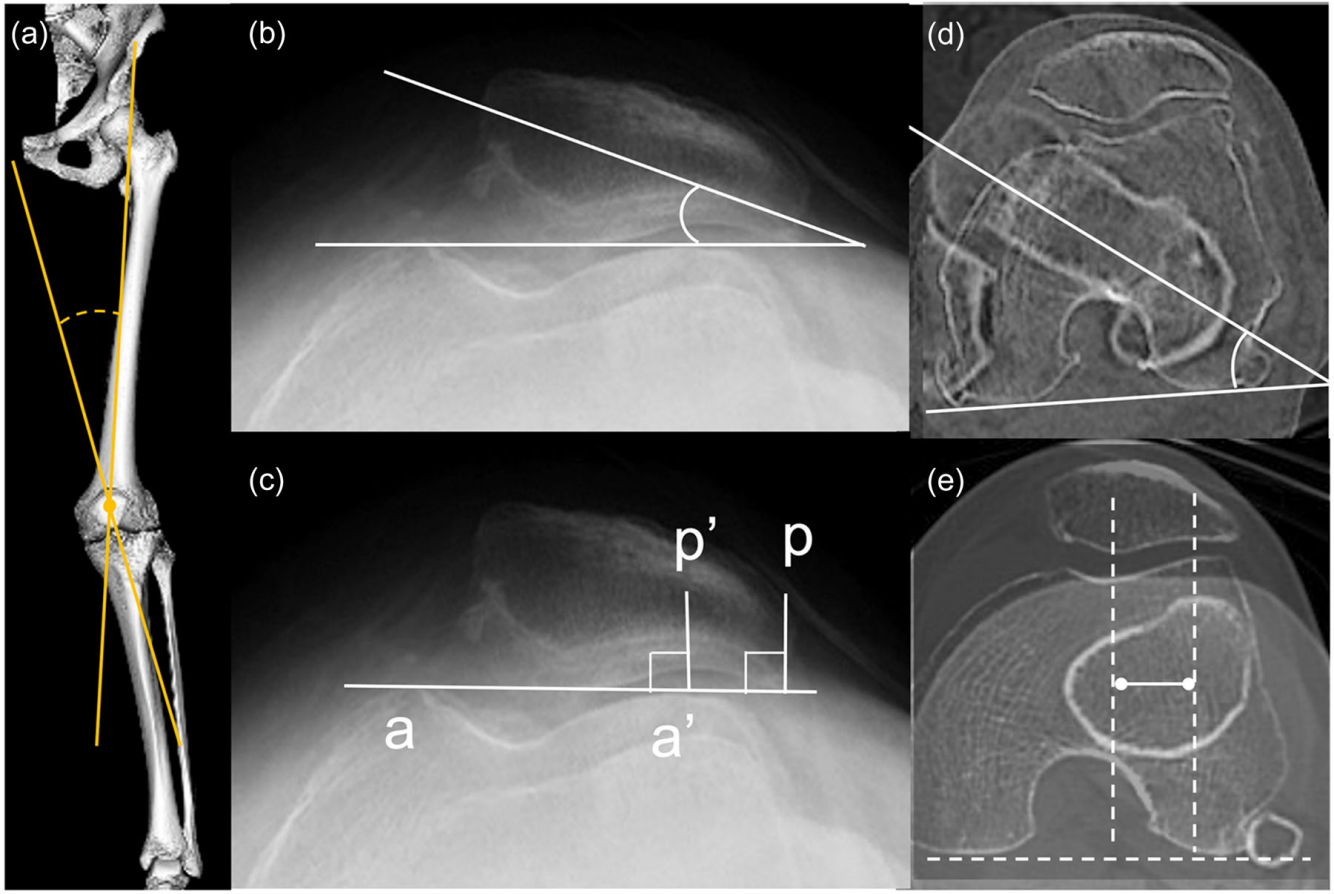
Radiographic assessments. (a) Radiological quadriceps angle (rQ angle) was measured as the acute angle between the line from the anterior superior iliac spine to the centre of the patella and the line from the tibial tuberosity to the centre of the patella. (b) Tilting angle was defined as the angle between the line intersecting the widest bony structure of the patella and the line tangentially passing the anterior surface of the femoral condyles. (c) Lateral shift ratio (LSR) was defined as the ratio of the distances pp′/aa′. aa′ is defined as the distance between the summits of the medial and lateral femoral condyles. pp′ is defined as the distance between the summit of the lateral femoral condyle and the point where a line from the lateral edge of the patella perpendicular to the line that passes through the summits of the femoral condyles crosses that line. (d) Femoral anteversion was measured as torsional angle of the femur between the line drawn parallel to the posterior femoral condyles and the line drawn through the centre of the femoral neck on the oblique axial CT. (e) Tibial tuberosity–trochlear groove (TT‐TG) distance was measured from two superimposed CT slices, one through the floor of the groove where the intercondylar notch has a Norman arch shape, and the other through the middle of the tibial tubercle. CT, computed tomography.

Radiological outcomes were appraised preoperatively and at the final follow‐up. OA severity in the FT and PF joints was classified using the KL classification, and OA progression was assessed by comparing serial postoperative images with preoperative baseline images to identify changes in joint space and osteoarthritic features. Measurements were conducted on a standing full‐leg anteroposterior radiographs and CT images. The HKA angle, representing the angle between the femoral and tibial mechanical axes; the lateral femorotibial angle (FTA), defined as the angle between the femoral and tibial shaft axes on the fibular side; and the mechanical lateral distal femoral angle (mLDFA), defined as the angle between the distal femoral joint line and the femoral MA, were assessed. The MA percentage was calculated as the horizontal distance from the MA to the medial edge of the tibial plateau, divided by the tibial plateau width. Patella height was evaluated using the Caton–Deschamps (CD) ratio on lateral radiographs [5], and the radiological quadriceps angle (rQ angle) was measured as the acute angle between a line from the anterior superior iliac spine to the patella centre and a line from the tibial tuberosity to the patella centre on CT (Figure 2a) [7, 10, 36]. PF joint alignment was evaluated via tilting angle and lateral shift ratio (LSR) on axial radiographs [20] (Figure 2b,c). Femoral anteversion was measured as the torsional angle between a line drawn parallel to the posterior femoral condyles and a line through the femoral neck on oblique axial CT [6] (Figure 2d), and tibial rotation relative to the femur was evaluated using the tibial tuberosity–trochlear groove (TT‐TG) distance [9] on CT (Figure 2e). All measurements were independently performed by two senior orthopaedic surgeons (M.M. and K.I.) with over 10 years of experience.

#### CT osteoabsorptiometry

Subchondral bone density across the trochlear and patellar PF surfaces was evaluated using CT osteoabsorptiometry before and more than 1 year after surgery. High‐resolution helical CT scans (Aquilion One/Vision Edition; Toshiba Medical Systems) were acquired with patients supine and knees extended, using 0.5‐mm slice thickness and intervals. DICOM and Communications in Medicine images were processed on a PC to generate sagittal and coronal slices at 1.0‐mm intervals and three‐dimensional (3D) bone models (Ziocube; Ziosoft, Inc.). Images were aligned along the distal femoral epicondylar axis, and subchondral bone of each compartment was manually delineated on sagittal, coronal and 3D PF images to encompass the entire articular surface [16].

Subchondral bone density in each sagittal slice was analysed with noncommercial software (OsteoDens 4.0) developed in‐house [14, 16, 24]. The region of interest (ROI) began at the point of maximal Hounsfield unit (HU) increment from the joint surface, with the maximum point in HU automatically selected within the 2.5‐mm ROI. Radiodensity was measured at 1‐mm intervals, producing two‐dimensional maps of subchondral bone density by stacking axial slices. HU values were categorized into nine grades, producing colour‐coded surface maps where red and violet represented the highest and lowest bone densities, respectively. Cortical bone and peripheral bony spurs were initially included due to software limitations but were carefully removed and manually excluded from the analysis target areas.

**Figure 3:**
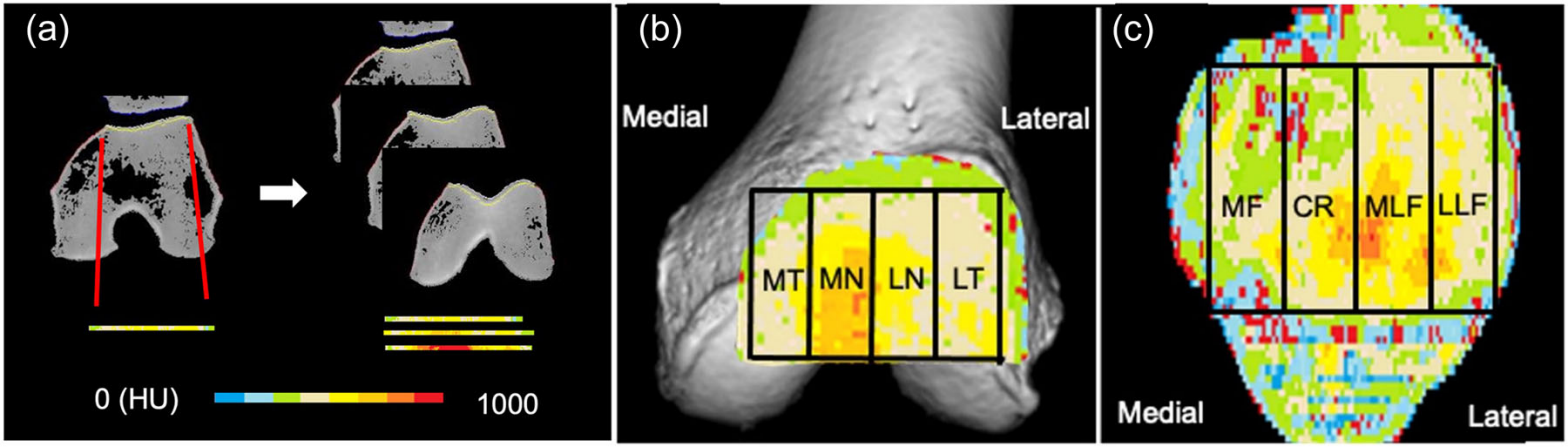
Identification of the subchondral bone regions in the patellofemoral (PF) articular surface using custom software. (a) Subchondral bone density of the selected region was automatically measured at each coordinate point in each 1.0‐mm sagittal slice. (b) Images representing the quantitative analysis areas used for bone density mapping in the trochlea. LN, lateral notch; LT, lateral trochlea; MN, medial notch; MT, medial trochlea. (c) Images representing the quantitative analysis areas used for bone density mapping in the patella. CR, central ridge; LLF, lateral portion of the lateral facet; MF, medial facet; MLF, medial portion of the lateral facet.

Quantitative analysis focused on the spatial distribution of HDAs across the articular surface. HDAs were defined as areas comprising the highest 30% HU values within each compartment. The lateral ratio was calculated as the proportion of HDA in the lateral two compartments relative to the total HDA across all four compartments. The percentage of HDA (%HDA) in each region was defined as the regional HDA divided by the total articular surface HDA [14, 16]. For the trochlea, regions included lateral trochlea (LT), the lateral notch (LN), the medial notch (MN) and the medial trochlea (MT). For the patella, regions included the lateral portion of the lateral facet (LLF), medial portion of the lateral facet (MLF), central ridge (CR) and medial facet (MF) [16] (Figure 3).

### Statistical analysis

Statistical analyses were performed using JMP Pro (version 16.0; SAS Institute, Inc.), with statistical significance set at *p* = 0.05. Means and 95% confidence intervals (CIs) were calculated for postoperative changes in patients with valgus knees. A priori power analysis (*α* = 0.05) was performed. Based on our previous studies [14, 16, 24], a sample size of 17 patients (17 knees) provided 80% power to test the hypothesis. Data normality was assessed using the Shapiro–Wilk test. Pre‐ and postoperative comparisons were analysed using a paired Student's *t* test. Effect sizes were calculated using Cohen's *d*. Pearson's correlation analysis was used to examine the association between the lateral ratio and other variables (age, body mass index [BMI], HKA angle, FTA, MA, mLDFA, rQ angle, tilting angle, LSR, TT‐TG distance and femoral anteversion) for preoperative patients. Furthermore, correlations between preoperative‐to‐postoperative changes (Δ) in the lateral ratio and corresponding changes in these variables were analysed using Pearson correlation. These variables were selected based on their established relevance to knee joint biomechanics and subchondral bone density distribution [12, 34]. Intra‐ and inter‐rater reliabilities for radiographic measurements were evaluated using intraclass correlation coefficients (ICCs). ICCs were calculated using a two‐way model with absolute agreement. ICC values were interpreted as poor (<0.50), moderate (0.50–0.75), good (0.75–0.90) and excellent (>0.90) reliability [21]. Using six randomly selected knees, the intra‐ and inter‐observer reliabilities of radiographic measurements were assessed by two examiners (T.O. and D.M.) at 1‐month intervals, with results presented in Supporting Information S1: Table S1. The reproducibility of measurements obtained with the noncommercial software (OsteoDens 4.0) was also assessed. Two observers (Y.S. and M.M.) independently measured the %HDA in six knees, with 48 subregions measured twice in a blinded manner at 1‐month intervals. The ICCs for intra‐observer reliability were 0.927 (excellent) (Y.S.) and 0.915 (excellent) (M.M.), while the ICC for interobserver reliability was 0.918 (excellent).

## RESULTS

### Patient characteristics and clinical outcomes

**Figure 4:**
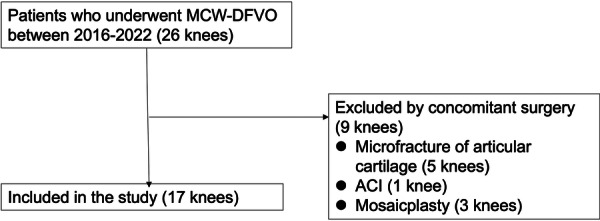
Study enrolment flowchart. ACI, autologous chondrocyte implantation; MCW‐DFVO, medial closing wedge‐distal femoral varus osteotomy.

This retrospective study initially enroled 26 consecutive patients (26 knees) who underwent MCW‐DFVO. Ultimately, 17 knees from 17 patients with a mean age of 48.4 years (range, 22–73 years, 14 females, 3 males) at the time of surgery were included in this study (Figure 4, Table 1).

**Table 1:** Patient characteristics and clinical outcomes (*n* = 17).

	Preoperative	Postoperative	*p* value	Effect size (Cohen's *d*)
Age, years	48.4 (16.9) (range: 22–73)			
Follow‐up duration, months		29.2 (range: 14–64)		
Male/female, *n*	3/14			
Body mass index, kg/m^2^	27.6 (5.6)			
Lysholm score, points	58.9 (19.6)	87.6 (7.1)	<0.001	0.812
The MCID responder rate for the Lysholm score, %	94.1 (16/17)			

*Note*: Data are reported as mean (SD) unless otherwise indicated. The MCID responder rate for the Lysholm score was reported as number of patients achieving MCID/total number of patients (%).

Abbreviations: MCID, minimal clinically important difference; SD, standard deviation.

Participant characteristics are detailed in Table 1. Clinical and radiological assessments were conducted preoperatively and at the final follow‐up (mean, 29.2 months; range, 14–64 months). Lysholm score showed significant improvement at the final follow‐up compared with preoperative values (*p* < 0.001 for each). The MCID responder rate for Lysholm score was 94.1% (16/17) (Table 1).

### Radiological and CT osteoabsorptiometry evaluations

The mean correction angle was 7.4°. Postoperatively, significant improvements were observed in the mean HKA angle, FTA, MA, mLDFA, rQ angle, tilting angle, LSR, femoral anteversion and TT‐TG distance (*p* < 0.001, *p* < 0.001, *p* < 0.001, *p* < 0.001, *p* = 0.07, *p* = 0.013, *p* < 0.001, *p* = 0.046 and *p* = 0.005, respectively). Moreover, the mean lateral ratio of trochlea and patella significantly decreased from 68.9% to 50.1% and from 68.6% to 56.5% (*p* = 0.004 and *p* = 0.041, respectively). Radiological evaluations postoperatively showed no OA progression in the FT and PF joints (Table 2).

**Table 2:** Radiological evaluations.

	Preoperative	Postoperative	*p* value	Effect size (Cohen's *d*)
Correction angle, degree	N/A	7.4 (1.5)		
HKA angle, degree	6.3 (4.9)	−1.9 (3.0)	<0.001	0.883
FTA, degree	168.2 (4.3)	176.6 (3.6)	<0.001	0.891
MA, %	74.6 (19.5)	42.1 (11.7)	<0.001	0.833
mLDFA, degree	81.7 (4.0)	89.3 (3.5)	<0.001	0.779
FT OA, no. of knees				
Grade 2	6	6		
Grade 3	11	11		
Lateral ratio of trochlea, %	68.9 (18.5)	50.1 (17.5)	0.004	0.591
Lateral ratio of patella, %	68.6 (21.0)	56.5 (20.8)	0.041	0.437
CD ratio, %	0.94 (0.3)	0.93 (0.2)	0.271	0.049
rQ angle, degree	12.5 (4.6)	8.1 (4.4)	0.007	0.572
Tilting angle, degree	9.4 (4.3)	7.4 (3.3)	0.013	0.523
LSR, %	18.2 (6.2)	13.7 (5.1)	<0.001	0.723
Femoral anteversion, degree	23.1 (11.6)	20.6 (11.4)	0.046	0.424
TT‐TG distance, mm	13.5 (8.8)	11.6 (9.2)	0.005	0.515
Patellofemoral OA				
Grade 1	3	3		
Grade 2	8	8		
Grade 3	6	6		

*Note*: Data are reported as mean (SD) unless otherwise indicated.

Abbreviations: CD ratio, Caton–Deschamps ratio; FT, femorotibial; FTA, lateral femorotibial angle; HKA, hip–knee–ankle angle; LSR, lateral shift ratio; MA, mechanical axis; mLDFA, mechanical lateral distal femoral angle; N/A, unavailable; OA, osteoarthritis; rQ angle, radiological quadriceps angle; SD, standard deviation; TT‐TG distance, tibial tuberosity to trochlea groove distance.

Pearson correlation analysis demonstrated significant correlations between the lateral ratio of the trochlea and the rQ angle and LSR (*p* = 0.036 and *p* = 0.011, respectively) (Table 3 and Supporting Information S1: Figure S1). However, none demonstrated a significant correlation with the lateral ratio of patella (Table 3).

**Table 3:** Pearson correlation between the lateral ratio of trochlea and patella and patient data before surgery.

	Lateral ratio of trochlea		Lateral ratio of patella	
	*r* value	*p* value	*r* value	*p* value
Age	−0.228	0.432	0.405	0.150
Body mass index	−0.268	0.352	−0.435	0.119
HKA angle	−0.303	0.291	0.248	0.392
FTA	0.011	0.969	0.223	0.441
MA	−0.115	0.695	−0.377	0.183
mLDFA	−0.181	0.522	0.093	0.750
CD ratio	−0.099	0.734	−0.298	0.300
rQ angle	0.509	0.036	0.249	0.389
Tilting angle	−0.001	0.997	−0.214	0.461
LSR	0.596	0.011	0.150	0.607
Femoral anteversion	−0.059	0.840	−0.364	0.200
TT‐TG distance	0.375	0.183	−0.307	0.284

Abbreviations: CD ratio, Caton–Deschamps ratio; FTA, lateral femorotibial angle; HKA, hip–knee–ankle angle; LSR, lateral shift ratio; MA, mechanical axis; mLDFA, mechanical lateral distal femoral angle; rQ angle, radiological quadriceps angle; TT‐TG distance, tibial tuberosity to trochlea groove distance.

Among patient characteristics, including age and BMI none demonstrated a significant correlation with the change in the lateral ratio of the trochlea (Δlateral ratio of the trochlea) (Supporting Information S1: Table S2). However, among the parameters altered after MCW‐DFVO, the HKA angle, MA, rQ angle and LSR showed significant correlations with the Δlateral ratio of the trochlea (*p* = 0.027, *p* = 0.031, *p* = 0.024 and *p* = 0.008, respectively) (Table 4).

**Table 4:** Pearson and partial correlation coefficients between the Δlateral ratio of trochlea and patient data in patients with valgus knees before and after MCW‐DFVO.

	Correlation coefficient	*p* value	Partial correlation coefficient
ΔPre‐ versus post‐DFVO			
HKA angle	0.585	0.027	0.208
MA	0.574	0.031	0.272
rQ angle	0.597	0.024	0.361
LSR	−0.671	0.008	0.483

Abbreviations: DFVO, distal femoral varus osteotomy; HKA, hip–knee–ankle angle; LSR, lateral shift ratio; MA, mechanical axis; MCW, medial closing wedge; rQ angle, radiological quadriceps angle.

Subregional %HDA analysis of the femoral trochlea revealed that there was a significant increase in the %HDA of the MT and MN regions in the trochlea postoperatively (*p* = 0.001 and *p* = 0.005, respectively). Conversely, the %HDA in the LN and LT regions of the trochlea significantly decreased postoperatively (*p* = 0.005 and *p* = 0.015, respectively). Moreover, the %HDA in the CR regions of the patella significantly increased postoperatively (*p* = 0.043). Conversely, the %HDA in the LLF regions of the patella significantly decreased postoperatively (*p* = 0.046) (Table 5).

**Table 5:** Subregional analysis of percentage high‐density area.

Subregion	Preoperative	Postoperative	*p* value	Effect size (Cohen's *d*)
MT	10.8 (7.1)	21.8 (7.1)	0.001	0.731
MN	20.1 (8.8)	27.9 (7.9)	0.005	0.632
LN	39.1 (6.6)	29.4 (5.5)	0.005	0.619
LT	29.7 (11.9)	20.6 (7.9)	0.015	0.585
MF	11.9 (6.5)	14.9 (9.5)	0.209	0.124
CR	19.3 (6.9)	26.5 (8.9)	0.043	0.431
MLF	37.1 (8.8)	34.1 (11.2)	0.073	0.193
LLF	31.5 (7.2)	24.3 (9.8)	0.046	0.425

*Note*: Data are reported as the mean (standard deviation).

Abbreviations: CR, central ridge; LLF, lateral portion of the lateral facet; LN, lateral notch; LT, lateral trochlea; MF, medial facet; MLF, medial portion of the lateral facet; MN, medial notch; MT, medial trochlea.

## DISCUSSION

The present study demonstrated that after MCW‐DFVO, the mean lateral ratio of the trochlea and patella significantly decreased. Second, the changes in the lateral ratio of the trochlea after MCW‐DFVO were significantly correlated with changes in the HKA angle, MA, rQ angle and LSR. Additionally, MCW‐DFVO markedly reduced the %HDA in the LN and LT regions, indicating a notable shift of stress distribution from the lateral to the medial PF articular surface.

Reports differ on whether concomitant PF OA should be considered a contraindication for MCW‐DFVO, with some citing it as unsuitable [37, 40], and others as an appropriate indication [34, 39]. Furthermore, additional procedures, such as medial PF ligament reconstruction [2] or tibial tuberosity advancement [8], have also been performed for patellar instability alongside MCW‐DFVO. In this study, MCW‐DFVO alone reduced the tilting angle, LSR and TT‐TG distance, improving PF joint congruence and suggesting that MCW‐DFVO may be beneficial, even in cases of PF OA.

Previous studies [1, 31] have suggested that DFVO might improve patellar tracking and PF joint congruence by reducing the Q‐angle in valgus knee. Consistent with this, our study found significant decreases in the rQ angle, tilting angle and LSR. The medial tilt of the patella occurred because of a medial shift of the tibial tuberosity, elevating the medial side of the trochlea and patella. These results indicate that MCW‐DFVO likely shifts the subchondral bone density distribution across the PF articular surface from the lateral to the medial PF articular surface.

Liska et al. [22] reported that reducing the anterior femoral torsion angle increases medial PF joint contact pressure while decreasing lateral pressure. Other studies have also shown that osteotomy to adjust femoral torsion can improve PF congruity [7, 13]. In the present study, a significant decrease in the anterior femoral torsion angle was observed, which likely contributed to the reductions in the tilting angle and LSR. Additionally, previous reports have noted a decrease in TT‐TG distance after DFVO [31, 32], and Imhoff et al. [13] reported a correlation between femoral torsion and TT‐TG. Thus, the observed changes in femoral torsion may have contributed to the reduced TT‐TG distance. This study found that ΔHKA angle, ΔMA, ΔLSR and ΔrQ angle were significantly correlated with the change in lateral ratio, highlighting the substantial impact of leg and PF alignment on PF joint stress distribution.

This study has several limitations. First, knee stresses were not measured directly but inferred from subchondral bone density using CT osteoabsorptiometry [14, 16], so the absolute stress at each point remains undetermined and may not reflect actual loading. Second, the wide age range of participants (22–73 years) could have influenced the outcomes due to age‐related variations in bone quality. Given the substantial differences in age, activity level and pathology, direct comparison of absolute values in this small sample may be misleading. Notably, age did not correlate with the lateral ratio, whereas leg alignment was significantly associated with the lateral ratio, suggesting that valgus alignment may have a stronger influence on subchondral bone density distribution than age. Third, the study's small sample size (17 knees) limits the generalizability of the findings. The strict inclusion criteria, excluding patients with meniscal injuries, may have biased the results toward patients with positive outcomes. Inclusion and exclusion criteria [3] were established to accurately evaluate the sole influence of MCW‐DFVO on subchondral bone density distribution. The results apply to a highly selected subgroup of valgus knees and may not be generalizable to typical valgus OA populations. Fourth, postoperative evaluations were performed within a relatively short time frame (mean, 29.2 months; range, 14–64 months), which may not fully capture the long‐term effects of stress distribution changes in the knee. In addition, long‐term clinical outcomes remain limited, restricting assessment of the lasting efficacy of the surgical intervention. Fifth, the interval between surgery and follow‐up varied widely (14–64 months). Although subchondral BMD remodelling occurs over extended periods, the mean time to postoperative radiograph and CT absorptiometry imaging was 14.6 months (range, 13–21 months), suggesting that HDA may not have reached a final steady state. Sixth, clinical outcomes were not assessed using a standardized PF‐specific symptom scale, such as the Kujala anterior knee pain score, limiting insight into the relationship between subchondral bone density changes and patient‐reported PF symptoms.

The findings of this study have important clinical implications for the management of valgus knees undergoing MCW‐DFVO. MCW‐DFVO not only restores coronal alignment and improves clinical outcomes but also modifies PF joint stress distribution. The observed medial shift of subchondral bone density suggests a reduction in lateral PF loading following surgery. These findings indicate that MCW‐DFVO may be considered in patients with valgus alignment and concomitant PF mal‐tracking or lateral PF overload, without the need for additional PF realignment procedures in appropriately selected cases.

In conclusion, this study showed that MCW‐DFVO shifted HDAs from the lateral to the medial PF articular surface, and the degree of leg and PF alignment correction post‐MCW‐DFVO was closely associated with these changes, reflecting changes in stress distribution.

## AUTHOR CONTRIBUTIONS

*Study conception and design*: Eiji Kondo, Masanari Hamasaki and Norimasa Iwasaki. *Acquisition of data*: Masanari Hamasaki, Masayuki Inoue, Kazunori Yasuda, Daisuke Momma and Tomonori Yagi. *Analysis and interpretation of data*: Masanari Hamasaki, Yuki Suzuki, Masatake Matsuoka and Koji Iwasaki. *Drafting*: Eiji Kondo, Masanari Hamasaki, Kazunori Yasuda, Tomohiro Onodera and Norimasa Iwasaki. All authors contributed significantly to this research and have seen and approved the contents of manuscript.

## CONFLICT OF INTEREST STATEMENT

The authors declare no conflicts of interest.

## ETHICS STATEMENT

This study protocol was approved by Hokkaido University Hospital Institutional Review Board (017‐0163), and each participant provided informed consent.

## Supporting information

Supplementary Information.

## Data Availability

All data generated or analysed during this study are included in this article. The datasets used and analysed during the current study are available from the corresponding author on reasonable request.
