# Supplementary material for: Medial closing wedge distal femoral varus osteotomy alters the stress distribution pattern of the patellofemoral joint: An evaluation using computed tomography osteoabsorptiometry
Source: J Exp Orthop. 2026 Apr 20;13(2):e70689. doi: 10.1002/jeo2.70689 (PMC13094811; doi:10.1002/jeo2.70689)
Supplement: Supplementary file 1 — Supplementary Information. [file JEO2-13-e70689-s001.docx]

**Supplementary Information**

**Medial Closing Wedge Distal Femoral Varus Osteotomy Alters the Stress Distribution Pattern of the Patellofemoral Joint: An Evaluation Using Computed Tomography Osteoabsorptiometry**


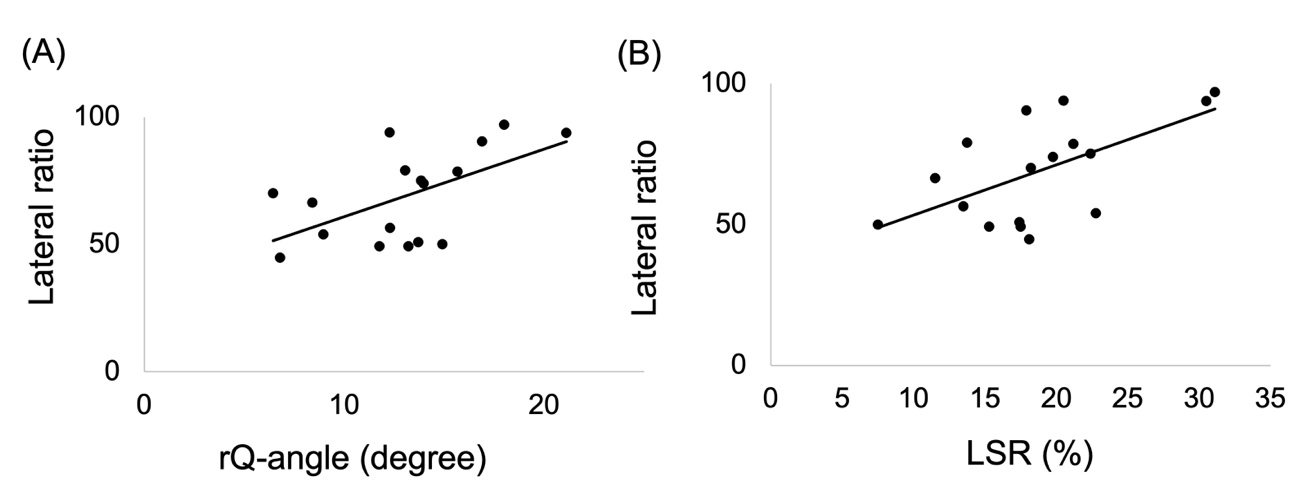


**Supplemental Figure 1.** (A) Relationship between the radiological quadriceps angle (rQ angle) and the lateral ratio of trochlea. Pearson correlation analysis shows a significant positive correlation between the rQ angle and the lateral ratio of trochlea (Pearson r = 0.509; *P*=0.036). (B) Relationship between the lateral shift ratio (LSR) and the lateral ratio. Pearson correlation analysis showed a significant positive correlation between the LSR and the lateral ratio of trochlea (Pearson r = 0.596; p=0.011). Lateral ratio: the ratio of the high-density area (HDA) of the medial compartment in relation to the total HDA of both compartments.

Supplemental TABLE 1.

Intraclass correlation coefficient scores for intra- observer and interobserver measurements

|  | Intra-observer reliabilities | | Interobserver reliabilities |
| --- | --- | --- | --- |
|  | Observer1 | Observer2 |  |
| HKA angle | 0.922 | 0.931 | 0.911 |
| FTA | 0.941 | 0.945 | 0.921 |
| MA | 0.937 | 0.931 | 0.921 |
| mLDFA | 0.922 | 0.933 | 0.912 |
| CD ratio | 0.856 | 0.897 | 0.873 |
| rQ angle | 0.918 | 0.942 | 0.937 |
| Tilting angle | 0.928 | 0.907 | 0.911 |
| LSR | 0.953 | 0.901 | 0.922 |
| Femoral anteversion | 0.911 | 0.857 | 0.884 |
| TT-TG distance | 0.899 | 0.887 | 0.878 |

HKA, hip–knee–ankle angle; FTA, lateral femorotibial angle; MA, mechanical axis; mLDFA, mechanical lateral distal femoral angle; rQ angle, radiological quadriceps angle; LSR, lateral shift ratio; TT-TG distance, tibial tuberosity to trochlea groove distance

Supplemental TABLE 2. Pearson and partial correlation coefficients between the Δlateral ratio of trochlea and patient data in patients with valgus knees before and after DFVO.

|  | Correlation Coefficient | *P* Value | Partial Correlation Coefficient |
| --- | --- | --- | --- |
| Pre-DFVO |  |  |  |
| HKA angle | −0.2929 | 0.309 | −0.182 |
| MA | 0.281 | 0.329 | −0.194 |
| LSR | 0.451 | 0.487 | 0.070 |
| rQ angle | 0.159 | 0.562 | −0.127 |
| Post-DFVO |  |  |  |
| HKA angle | −0.059 | 0.839 | −0.022 |
| MA | −0.047 | 0.872 | 0.095 |
| LSR | −0.017 | 0.951 | 0.028 |
| rQ angle | 0.179 | 0.538 | 0.171 |

HKA, hip–knee–ankle angle; FTA, lateral femorotibial angle; MA, mechanical axis; mLDFA, mechanical lateral distal femoral angle; lateral ratio, ratio of the high-density area (HDA) of the medial compartment to the total HDA of both compartments.
